# European Society of Paediatric Radiology 2019 strategic research agenda: improving imaging for tomorrow’s children

**DOI:** 10.1007/s00247-019-04406-4

**Published:** 2019-05-21

**Authors:** Owen J. Arthurs, Rick R. van Rijn, Claudio Granata, Luciana Porto, F. Wolfgang Hirsch, Karen Rosendahl

**Affiliations:** 1 Department of Radiology, Great Ormond Street Hospital, London, UK; 2 NIHR UCL Great Ormond Street Institute of Child Health Biomedical Research Centre, London, UK; 3 Department of Radiology, Emma Children’s Hospital – Amsterdam UMC, University of Amsterdam, Amsterdam, The Netherlands; 4 Service of Radiology, IRCCS Istituto Giannina Gaslini, Genoa, Italy; 5 Department of Paediatric Neurology, Hospital of Goethe University, Frankfurt am Main, Germany; 6 Department of Paediatric Radiology, University Leipzig, Leipzig, Germany; 7 Department of Radiology, Haukeland University Hospital, 5020 Bergen, Norway; 8 Department of Clinical Medicine, University of Bergen, Bergen, Norway

**Keywords:** Guidelines, Imaging, Paediatric, Research

## Abstract

The European Society of Paediatric Radiology (ESPR) research committee was established to initiate, drive forward and foster excellence in paediatric imaging, paediatric image-guided intervention and radiation protection research, by facilitating more evidence-based standards, protocols and multi-institutional collaborations. The ESPR Strategic Research Agenda outlines our current research approach, highlighting several areas of paediatric imaging where the society can help guide current and future research, and emphasizing those areas where early research (“seed”) funding may need to be allocated by this and other societies as precursors to larger grant applications. The key aims are to evaluate normal variation in order to be able to confidently diagnose disease states, develop robust image-based classification systems to aid diagnosis and treatment monitoring, and help develop evidence-based clinical guidelines using current literature and experience to identify knowledge gaps. For this reason, the development of evidence-based imaging pipelines, broken down step-by-step to include diagnosis, classification and clinical effectiveness, should be the end goal for each disease entity for each affected child. Here, we outline the 2019 ESPR Strategic Research Agenda along three points in the clinical imaging pipeline: clinical referral, disease diagnosis and evolution, and clinical therapeutic evaluation and effectiveness. Through multicentre trials, using existing high-level experience and expertise, and nurturing the next generation of researchers, we will be able to achieve these aims.

## Introduction

Paediatric radiology is the skill of accurate and appropriate imaging of childhood disease, a key component being safety [1, 2]. The European Society of Paediatric Radiology (ESPR) strives that all children should be examined and diagnosed by specialists with appropriate expertise, using the best possible technology available. Ideally, therefore, there should be round-the-clock paediatric radiology expertise available either on-site or via a clinical network of specialists.

Whilst several countries have dedicated large children’s hospitals with specialist radiology staff with paediatric skills, much of paediatric imaging is undertaken by nonspecialists. The most recent publication from the Royal College of Radiologists in the United Kingdom estimates that 75% of children’s radiographs and scans are taken in smaller, nonspecialist hospitals [3], by radiographers who have no specific training in imaging children, and interpreted by radiologists with less than 6 months’ specialist training. Although the technology for transferring images is now widely available, the majority of centres (62% in the United Kingdom, 90% in Norway, for example) still do not have access to a 24-h paediatric opinion [4, 5]. Across Europe, many paediatric examinations are therefore reported by general radiologists, or non-radiology clinicians.

The importance of this child-centric and child-specific approach has been addressed in several recent publications showing a significant rate of major disagreements between interpretations of paediatric imaging studies by general radiologists and those of specialty radiologists at a tertiary care paediatric hospital [5, 6]. Notably, there was a significant correlation between the second opinion interpretations and the final diagnoses, thus assuming a crucial role in patient management. These findings are similar to studies on specialist reporting in different adult populations and underline the importance of subspecialty training [7–12]. The results are alarming and should fuel our efforts to enhance education and research within paediatric imaging.

By strengthening research in paediatric radiology, we will increase the impact of our subspecialty and form a research basis for the foundation for paediatric radiology clinical practice. With this in mind, the ESPR research committee was established to initiate, advance and foster excellence in paediatric imaging, including paediatric image-guided intervention and radiation protection research. It would do this by facilitating the progression away from individual, locally isolated projects toward more evidence-based standards, protocols and multi-institutional collaborations, which could be used on both a national and international basis.

The ESPR research committee has identified several areas of paediatric imaging for essential research, but until now has not had a formal Strategic Research Agenda under which to coordinate these projects. This document acts not only as a strategic agenda for the society to help guide current and future research, ensuring that it aligns closely with wider European Society of Radiology (ESR)’s European Institute for Biomedical Imaging Research and European commission funding calls, but also to highlight those particular areas where early research (“seed”) funding may need to be allocated by this and other societies as precursors to larger grant applications. Through multicentre trials, existing high-level experience and expertise, and nurturing the next generation of researchers, we will be able to achieve these aims. This research agenda is mission orientated and impact focused, and necessitates the free movement of data among researchers. In order to achieve our aim, the ESPR research committee underwrites the policy in which research data are Findable, Accessible, Interoperable, and Reusable (FAIR) [13].

Research that can clearly measure or emphasize the advantages of specialized imaging is the future of our specialty. Investment in well-designed research trials to address today’s challenges and questions will improve the health care of tomorrow’s children. Clearly, all resources are finite, so time and funding should be prioritized to the most urgent and far-ranging problems. Areas of current study that warrant further work include non-oncological bowel imaging in children, management of radiation exposure in paediatric imaging and the application of advanced new imaging methods to complex paediatric disease.

The key aims of the ESPR research committee are to:

1. Improve imaging-based paediatric research through multicentre trials and collaborative working, including data sharing;
2. Document normal variation in imaging findings to be able to confidently differentiate disease from normal;
3. Develop robust image-based classification systems to aid diagnosis and treatment monitoring, and
4. Help develop evidence-based clinical guidelines using current literature and experience to identify knowledge gaps.

## Overarching strategy

Evidence-based clinical imaging pathways are the ultimate end point of successful imaging research as they provide an efficient system of the maximum and minimum imaging requirements to make a diagnosis in a particular clinical situation, using the best available literature at the time. If implemented correctly, they also allow for a reduction in practice variation leading to overall improvement in clinical care. For this reason, the development of evidence-based imaging pipelines, broken down step-by-step to include diagnosis, classification and interval monitoring, should be the end goal for each disease entity for each child.

**Fig. 1:**
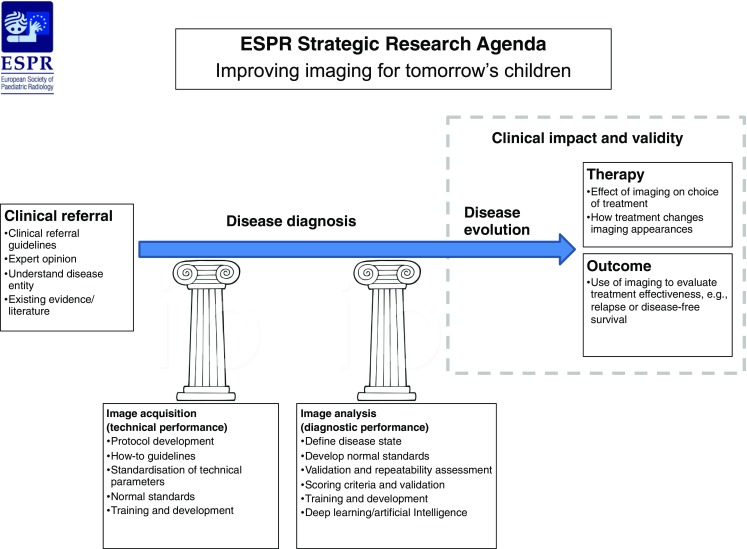
European Society of Paediatric Radiology 2019 Strategic Research Agenda: Improving imaging for tomorrow’s children. The diagram outlines the pipeline from clinical referral to disease diagnosis to disease evolution. Disease diagnosis is underpinned by the two pillars of image acquisition (technical performance) and image analysis (diagnostic performance). Ultimately, only clinically impactful and effective imaging should be employed

The three steps in any imaging pipeline, loosely based on Fryback and Thornbury framework [14], are (Fig. 1):

- Clinical referral and diagnostic criteria: risk factors and clinical presentation.
- Disease diagnosis and evolution: image acquisition and analysis.
- Clinical impact: therapeutic evaluation and clinical effectiveness.

### Clinical referral and diagnostic criteria

The appropriate imaging for the correct clinical scenario is still led by experience rather than evidence. In many cases, imaging algorithms have developed through patient and clinician experience and expertise without being validated through comprehensive studies. Clinical imaging referral guidelines play a fundamental role in enhancing appropriateness and thus the implementation of the principle of justification. Modern systematic reviews and meta-analyses provide a critical review of the existing literature and can provide much needed insight, but expert opinion is often the resource used when evidence is lacking. Literature studies or questionnaires also help to highlight current knowledge and practice gaps, and can initiate new research projects.

The ESPR recognizes the value of expert opinion by supporting several imaging task forces that have been active in evaluating the literature within their areas of expertise and generating consensus documentation. Here, the ESPR abdominal (gastrointestinal and genitourinary) task force can serve as an excellent example. Over the past 10 years, in close collaboration with other international societies such as the European Society of Uroradiology, the task force has provided imaging recommendations that can be implemented in daily practice [15, 16]. These task force recommendations are preferentially published in the society journal *Pediatric Radiology*. Several of these have become implemented as paediatric imaging referral guidelines in the recent ESR iGuide clinical decision support system for referrers and radiologists [17].

### Disease diagnosis and evolution

#### Image acquisition

One of the fundamental aspects of paediatric imaging research is the ability to be able to share data sets among centres, particularly in cases of rare disease. However, the inability to standardize image acquisition (operating protocols and specific sequence acquisitions) hampers the ability to analyse comparable data across institutions, occasionally resulting in additional imaging tests being performed. It is for this reason that the standardization of technical parameters is paramount going forwards, but also perhaps the largest hurdle to overcome. This challenge occurs on many levels, includes the standardization of imaging approaches (e.g., the use of ultrasound [US] in cystic kidney disease [18]), imaging protocols (e.g., the use of gadolinium for joint disease activity assessment in juvenile idiopathic arthritis [19]), and further specific sequences within imaging protocols (e.g., the use of diffusion-weighted imaging in oncology assessment as routine [20]).

Paediatric oncology and musculoskeletal imaging are good examples of how this has been achieved through international guidelines, but this is lacking in several other areas. For example, the combined efforts of the OMERACT (Outcome Measures in Rheumatology) and Health e-Child have developed working guidelines for imaging of the wrist and knee in juvenile idiopathic arthritis [21–23]. Within the European Paediatric soft-tissue sarcoma study group, there has been strong involvement of paediatric radiologists and nuclear physicians with respect to the European Frontline and Relapsed-RhabdoMyoSarcoma Study (FaR-RMS). Minimum clinical imaging protocols have been proposed and accepted, and participating centres will use the Quality and Excellence in Radiotherapy and Imaging for Children and Adolescents with Cancer across Europe in Clinical Trials (QUARTET) network to share imaging. This allows for both expert reading and multicentre-multinational research. Similar initiatives have been established across a range of children’s cancers, such as the Society for Pediatric Oncology brain tumour imaging group, encouraging the translation of new imaging methods into clinical trials to assess their effectiveness.

Decisions regarding which imaging modality is best to answer which clinical question often involve trade-offs between ease of access to imaging types, radiation dose and the level of detail required. This is all the more difficult when studies are not only multicentre but also multinational, as there is unequal access to more advanced imaging techniques (e.g., high field magnetic resonance imaging [MRI] or positron emission tomography) between centres and countries. Meanwhile, reducing the radiation dose of ionizing radiation modalities (radiography, fluoroscopy, computed tomography, nuclear medicine) remains a priority, while maintaining or improving image quality [24]. Improving the quality and efficiency of modalities that do not use ionizing radiation (US, MRI), for example improving MRI to make it faster, more capable and potentially avoiding sedation or anaesthesia, is clearly a priority [2, 25]. Image acquisition strategies in children must evaluate dose in two ways: by improving dose efficiency in studies in which ionizing radiation cannot be avoided, but also by improving non-radiation-based imaging techniques.

Good examples of the significant work done across Europe in this regard include the ESPR’s pivotal role in the PiDRL project (European Diagnostic Reference Levels for Pediatric Imaging; http://www.eurosafeimaging.org/pidrl). This multi-partner European Consortium-lead project headed by the ESR was intended to provide European Diagnostic Reference Levels (DRLs) for paediatric examinations. By doing so, their use would be promoted to advance the optimization of radiation protection of paediatric patients, with a focus on CT, interventional procedures using fluoroscopy, and digital radiographic imaging. Their first steps were to agree on a methodology for establishing and using dose reference levels for paediatric imaging, and to update and extend the European DRLs to cover more procedures and a wider patient age/weight range. The final document including European guidelines on dose reference levels for paediatric imaging has been endorsed and published by the European Commission and is available in the Radiation Protection Series [26].

However, more work is needed. The International Commission on Radiological Protection (ICRP) advises that clinical indication-specific, rather than examination-specific, dose reference levels are desirable. For example, CT examinations of the same anatomical regions can be performed with different techniques, and consequently different dose exposures, depending on the clinical indication. However, there is very limited information about clinical-indication specific dose reference levels for medical imaging in children. The next project to establish new European clinical dose reference levels in children is long awaited.

#### Image analysis

Once image acquisition standardisation is achieved, simultaneously there must be image analysis standardisation, i.e. of classifications, measurements and scoring systems. Key priorities for image analysis (or assessment of diagnostic performance) are to develop robust methods of diagnosing a specific disease state, developing normal standards that stand up to rigorous testing, validation and repeatability assessment, and classification or scoring criteria with validation.

Normal reference appearances and values are essential to correctly interpret diagnostic images, particularly in children as normal appearances change during growth. The ability to distinguish normal variations from abnormal disease in its earliest form is the cornerstone of paediatric imaging, but this is largely experiential rather than evidential in its current practice, leading to wide variations in interpretation without evidence to the contrary. All trainee radiologists will be familiar with Keats’ standard textbook of normal variants which mimic disease, but the “evidence” behind this book is experiential [27]. Normal standards are frequently compiled into an atlas, against which specific imaging phenotypes can be assessed. Some reference standards are becoming available for normal variations in specific clinical scenarios, for example the reference standards for kidney size [28, 29]. There are no normal standards for whole-body MRI, which poses particular problems for diagnosing abnormalities [30]. In other areas of musculoskeletal imaging, recent advances have been made regarding image-based classification systems for juvenile dermatomyositis [31] and establishment of normal US-based references for the wrist [32].

Areas for future study would include wide variation of ventricular size in healthy newborns and young children, which is crucial knowledge to identify hydrocephalus [33], or the evaluation of normal bowel wall thickness to evaluate inflammatory bowel disease. Collaborative cohort studies including paediatric radiologists are clearly needed: for example, a prospective cohort study from fetal life until young adulthood in a multiethnic urban population based in Rotterdam (known as the Dutch Generation R study) that is using long-term imaging as part of assessments (https://www.generationr.nl/researchers/data-collection/).

### Clinical impact: Therapeutic evaluation and clinical effectiveness

Many of the aspects of image acquisition and analysis apply equally, whether it is the first or last scan in a series of complex patient interactions. But there are more questions than answers to be explored here: When to image? What is the appropriate time interval to image in certain diseases? What are the risks in doing so (missing early disease states or relapse) versus those of imaging too early (latency bias)? Imaging is frequently used to evaluate disease evolution despite a lack of evidence for its clinical utility. Accurate imaging markers of effective therapy are essential for disease reassessment to be worthwhile. Imaging is frequently used in an attempt to evaluate a patient’s response to therapy, whether pharmacological or surgical. Whilst it is relatively straightforward to image and observe change associated with treatment, proving that a change has occurred outside of normal variation and that it is causally linked to the treatment is not always clear. Clinical improvement may not correspond to imaging changes, and vice versa, or may be temporally displaced. Worse still, imaging may identify incidental lesions with the risk of overtreatment. How accurately we can predict disease monitoring is challenging, and imaging frequency may need to be tailored to individual disease states and risk factors.

Imaging is heavily used in paediatric oncology assessment and reassessment, and has already demonstrated problem areas. For example, imaging following tumour resection can be challenging: Marginal amounts of residual tumours may not be visible using current imaging techniques, postsurgical contrast enhancement around resection margins can be physiological or reactive and they do not necessarily infer residual tumour. Reduction of tumour volume of >50% following treatment may be termed “partial response,” but the exact nature of the residual tissue is currently not determinable.

Within the European paediatric soft-tissue sarcoma group, several recent retrospective studies have evaluated the impact of imaging findings on patient care, using rhabdomyosarcoma patients as the example group. One study showed that early radiologic response to chemotherapy (volumetric tumour reduction) did not infer a survival advantage in patients with rhabdomyosarcoma [34]. Another study showed that indeterminate lung nodules should be treated as non-metastatic in otherwise non-metastatic patients in rhabdomyosarcoma [35] and other studies have sought to evaluate the role of follow-up imaging [36–38]. Through evaluating the evidence for widely held beliefs, good collaborative paediatric radiologic research can clearly have a major clinical impact on treatment protocols and patient care.

## Examples of successful research along imaging pipelines

One example of a current and ongoing specific disease pipeline is the current pan-European research initiative into juvenile idiopathic arthritis (JIA). The Euro-Im-JIA, launched in 2013–14, addresses the current lack of imaging markers for JIA through developing precise, validated child-specific imaging biomarkers and scoring systems to allow for evidence-based clinical practice as well as for robust drug trials. The entire project is founded on multidisciplinary collaboration across several well-established research groups across Europe, including paediatric rheumatologists, radiologists, dentists and oral surgeons as well as medical physicists and mathematicians. This project proposes a standardisation of the assessment of active and permanent change in JIA, allowing better assessment of disease evolution and therapeutics [21, 23]. If successful, this work will allow earlier, more robust diagnosis, a more sensitive classification of potential for the development of pathology, and thus the opportunity for earlier intervention to reduce chronic change and disability. JIA is, of course, only one example of several childhood illnesses with lifelong consequences. Several other rheumatological conditions and hip problems (such as developmental hip dysplasia, slipped capital femoral epiphysis and femoral acetabular impingement) can all affect joints in childhood and adolescence, which may lead to accelerated degenerative disease in adulthood and the eventual need for hip replacement, often at a relatively young adult age. Imaging plays a key role in diagnosing these conditions, monitoring their progression and evaluating for complications, and thereby guiding therapy.

Cancer imaging is another such pipeline likely to improve in several domains for children’s services in the near future. Hybrid imaging is improving sensitivity across different imaging modalities, and may simultaneously improve specificity, particularly for metastatic disease [39, 40]. Newer cell-based imaging markers will become important in the future, not only to differentiate tumour subtypes at diagnosis, but also to target therapy and measure tumour responses. However, most cancer trials involving imaging are designed with imaging as an adjunct, or addition, to the main trial regarding new treatment paradigms or responses. This is true of many non-cancer imaging trials, which tend not to be led by paediatric radiologists but other specialists, such as oncologists or cardiologists. The ESPR is keen to foster imager-led research trials to improve the delivery of high-quality imaging, as well as to have radiologists play their part in larger clinical trials.

## Coordinated networks

Some of the data needed to be able to address these vital questions are already acquired and currently sit on disparate hospital networks across different countries and institutions. There are several issues with data access and coordinated studies, but multiuser sharing platforms such as those provided by the European Initiative in Biomedical Imaging Research, the QUARTET network, and the Dutch Health Research Infrastructure (Health RI (https://www.bbmri.nl/health-ri/) may help coordinate these studies in the future. The idea of a single imaging repository with anonymised access to vast amounts of verified clinical data is a long way from being possible with current technology and border controls, including industry, consent and ethical issues.

In the meantime, smaller national networks have developed successful collaborations with free data sharing within a strict research agreement and geographical distribution. There are many reference disease centres now established in different countries, providing care within and between nations. One function is to act as the liaison for imaging and clinical data access for European level projects, provided patients have consented for their data to be used in ethically approved multinational projects, with the necessary safeguards.

## Rare diseases

One of the most challenging and frustrating aspects of paediatric disease are rare diseases because research efforts are hampered by small numbers at each institution and often limited funding. Improved data transfer technology now allows better sharing of information across institutions, and universal patient medical records offer opportunities for improved collaboration on rare diseases, which will encourage more collaborative research efforts. However, as has already been described in standardisation, one obstacle to collaboration is heterogeneity in performing and reporting paediatric imaging. By agreeing to a set of standard methods on how to perform a certain test, the results will be easier to compare across different institutions, which will ultimately improve patient care.

## Conclusion

Imaging is paramount for efficient, high-quality paediatric medicine. Clinical and research pipelines to evaluate rare diseases will necessarily involve multiple aspects of this strategy, including establishing imaging protocols on the best diagnostic approaches, normal standards for phenotyping, imaging-based scoring systems to evaluate disease severity, clinical correlation with genetic and other biomarkers, and finally evaluation of potential treatments against these scoring systems and biomarkers.

Several of these initiatives (particularly dose reduction and optimisation, data registries and improving image analysis) are shared with other paediatric imaging representatives, such as the Paediatric Imaging Research Committee of the American College of Radiology (White paper [41]) and we look forward to joint initiatives.

Fostering clinical research collaboration at an international level is the ESPR research committee’s goal over the next 5 years to facilitate improvements in paediatric health care. Disease prevention and other aspects of health care have not been neglected nor will support for other initiatives be abandoned. These are simply the areas that we collectively believe as a society require the most intellectual and financial input in the next 5 years in order to develop imaging markers of disease that can be relied upon for diagnosis, monitoring and therapeutic evaluation. Fundamentally, we are investing in these priorities as the ESPR’s strategic agenda for 2019–2022. We look forward to the progress that will be made on these and other areas and to updating this research strategy in 3–5 years.
